# Morbidity patterns and long-term outcomes of central lymph node dissection in thyroid cancer patients

**DOI:** 10.1038/s41598-025-08439-8

**Published:** 2025-07-02

**Authors:** Khalid Atallah, Shadi Awny, Khaled Abdelwahab, Ahmed Abdallah, Islam H Metwally, Omar Hamdy, Mohammed Zuhdy, Ahmed Fareed

**Affiliations:** 1 Surgical Oncology Department, Oncology Center, Faculty of Medicine, Mansoura University, Mansoura, Egypt; 2 Surgical Oncology Department, King Abdullah Medical City, Makkah, Kingdom of Saudi Arabia

**Keywords:** Papillary thyroid carcinoma, Medullary thyroid carcinoma, Thyroid cancer, Central lymph node dissection, Hypocalcemia, Recurrent laryngeal nerve injury, Head and neck cancer, Cancer, Oncology, Surgical oncology

## Abstract

Central lymph node metastasis (CLNM) is observed in 20-90% of patients with differentiated thyroid cancer (DTC). Central lymph node dissection (CLND) is associated with a higher incidence of complications, including hypocalcemia and an increased risk of recurrent laryngeal nerve injury. This study aimed to characterize and analyze the morbidity of CLND precisely. This is a retrospective cohort study that included patients who were diagnosed with thyroid cancer and underwent total thyroidectomy and CLND at a tertiary cancer center from January 2012 to December 2023. Among 420 patients, intraoperative complications were observed in 47 patients (11.2%). The commonest was recurrent laryngeal nerve palsy, which occurred in 16 patients. Stridor was detected immediately after extubating five patients. The commonest postoperative complication was hypocalcemia, which occurred in 69 patients (16.4% of patients), 21 patients (5%) required hospital re-admission, and most of them were temporary (79.4%). CLND may lead to morbidity in 1 to 2 of every 10 patients. Better diagnostic tools and predictive models are mandatory to identify the central compartment disease burden. Moreover, more advanced surgical techniques are needed to spare the patients the potential complications.

**Supplementary Information:**

The online version contains supplementary material available at 10.1038/s41598-025-08439-8.

## Background

Thyroid cancer is the most prevalent endocrine malignancy. It accounts for 1% of all malignancies^1^. Papillary thyroid cancer (PTC) is the most common type, accounting for approximately 85–90% of all thyroid cancers, followed by follicular thyroid cancer (FTC), which accounts for 5–10%, while medullary thyroid cancer (MTC) accounts for about 4%^2,3^.

Central lymph node metastasis (CLNM) is observed in 20-90% of patients with differentiated thyroid cancer (DTC)^4^. The central compartment of the neck is well known as the first site of lymph node metastasis in both PTC and MTC, while CLNM is not common in FTC^5,6^.

The accuracy of neck ultrasonography in detecting CLNM is limited because paratracheal lymph nodes are minute and located beneath the thyroid gland^7^. For this reason, prophylactic central lymph node dissection (CLND) is frequently performed in patients with clinically negative central lymph nodes. It remains controversial whether elective or prophylactic CLND should be applied in patients with T1 and T2 cancer^3^.

On the other hand, total thyroidectomy with therapeutic CLND is recommended in patients with lymph node metastases in the neck identified on preoperative examination or at the time of surgery^8^.

CLND is associated with a higher incidence of complications. These complications include hypocalcemia that may be temporary or permanent (secondary to either devascularization or incidental removal of parathyroid glands during surgery) and increased risk of recurrent laryngeal nerve injury, leading to transient or permanent vocal cord palsy^9,10^.

In the literature, the definition of postoperative hypocalcemia varies widely; some researchers use biochemical data [calcium and parathyroid hormone (PTH) levels], while others focus on the presence of clinical symptoms requiring oral or intravenous (IV) calcium and vitamin D supplementation, the majority of patients with asymptomatic hypocalcemia need no treatment^11^.

Recurrent laryngeal nerve injury may be caused by intraoperative nerve injury due to either intended sacrifice of the nerve when infiltrated by the tumor, unintended iatrogenic injury, or due to temporary neuropraxia caused by edema or minor traction injury.

Therefore, this study aimed to precisely characterize the morbidity of CLND and analyze the predictive risk factors of relevant complications.

## Patients and methods

This is a retrospective cohort study. We included patients with thyroid cancer who were managed by total thyroidectomy and CLND with or without lateral lymph neck dissection (LLND) at Oncology Center, Mansoura University, from January 2012 to December 2023. We excluded patients who underwent total thyroidectomy alone without CLND, patients who underwent CLND in recurrent settings, and those in whom CLND did not reveal nodal tissue. Finally, 420 patients met these criteria and were included in our study. Demographics, preoperative, operative, postoperative, pathologic, and oncologic follow-up data were retrieved from a prospectively maintained electronic database.

This study aims to evaluate the morbidity of CLND in those patients and analyze the risk factors of these complications.

### Data

Based on our electronic database, we retrospectively analyzed both intraoperative and postoperative complications (mainly recurrent laryngeal nerve injury and hypocalcemia). Vocal fold function was assessed in all patients by indirect laryngoscopy before and after surgery. Recurrent laryngeal nerve injury was defined as dysfunction or total absence of vocal cord mobility compared to the contralateral one based on preoperative laryngoscopy. Nerve injury may be temporary or permanent. All vocal fold palsies lasting for more than 6 months were considered permanent. Postoperative serum calcium levels were measured only in patients with symptoms of hypocalcemia, while asymptomatic cases were not tested for hypocalcemia. Other relevant postoperative complications were reported, such as hematoma, chyle leakage, and other nerve damage (spinal accessory nerve, vagus nerve, phrenic nerve, and/or sympathetic trunk).

### Statistical analysis

We used the statistical software SPSS (Statistical Package for Social Sciences, SPSS 22.0; Armonk, NY: IBM Corp) to analyze the study results. Continuous variables were presented as mean and standard deviation if normally distributed or median and range when non normally distributed. An independent samples t-test was used to compare parametric data, whereas the Mann-Whitney U test was used to compare non-parametric data. Categorical data were compared by Pearson’s Chi-square test or the Fisher-Exact test when appropriate. A p-value of ˂0.05 was considered statistically significant.

## Results

### Demographics (Table 1)

**Table 1:** The demographic and operative data of our study.

Variable	Value
Age at surgery mean +/- SD years	35.5 +/- 15.4
BMI median (range) Kg/m2	31.1 (17.5–56.9)
Sex	
Male	118 (28.1%)
Female	302 (71.9%)
Radiologic laterality of malignant nodules	
Unilateral	270 (64.3%)
Bilateral	134 (31.9%)
Radiologic focality of malignant nodules	
Unifocal	209 (49.8%)
Multifocal	195 (46.4%)
Radiologic central node status	
Negative	363 (86.4%)
Positive	35 (8.3%)
Radiologic lateral node status	
Negative	200 (47.6%)
Positive	213 (50.7%)
Type of nodal surgery	
Ipsilateral CLND	125 (29.8%)
Bilateral CLND	57 (13.6%)
Ipsilateral CLND & LLND	127 (30.2%)
Bilateral CLND and ipsilateral LLND	50 (11.9%)
Bilateral CLND and LLND	46 (11%)
Ipsilateral CLND and bilateral LLND	15 (3.6%)
Pattern of nodal dissection	
Prophylactic	128 (30.5%)
Therapeutic	227 (54%)
Sampling	40 (9.5%)
Excised with specimen	16 (3.8%)
Number of retrieved central nodes median (range)	5 (1–35)

Four hundred and twenty patients were recruited, with female predominance (302 patients). The mean age at diagnosis was 35.5 years. All the patients underwent CLND. More than half of them (54%) were therapeutic, while the rest were either prophylactic (formal dissection in 30.5% and sampling in 9.5%) or excised incidentally with the total thyroidectomy specimen.

The median number of retrieved central nodes was 5 (1–35). Ipsilateral CLND and LLND were done in 127 patients (30.2%), and ipsilateral CLND only was carried out in 125 patients (29.8%). While bilateral CLND *without* LLND was done in 57 patients (13.6%).

### Pathology (Table 2)

**Table 2:** The pathologic data.

Variable	Value
Pathologic type	
Papillary thyroid cancer	389 (92.6%)
Medullary thyroid cancer	21 (5%)
Follicular thyroid cancer	5 (1.2%)
Hurthle cell cancer	1 (0.2%)
Anaplastic thyroid cancer	1 (0.2%)
Spindle cell tumor with thymus-like differentiation (SETTLE)	1 (0.2%)
Pathologic focality	
Unifocal	211 (50.2%)
Multifocal	196 (46.7%)
Pathology laterality	
Unilateral	269 (64%)
Bilateral	140 (33.3%)
Extrathyroid extension	
No	244 (58.1%)
Yes	111 (26.4%)
Pathologic Central node status	
Negative	141 (33.6%)
Positive	274 (65.2%)
Lateral node status	
Negative	30 (12.7%)
Positive	207 (87.3%)
T stage	
1	147 (35%)
2	138 (32.9%)
3	102 (24.3%)
4	17 (4%)
N stage	
0	116 (27.6%)
1a	94 (22.4%)
1b	207 (49.3%)

Valid percent*.

PTC was the most common pathology, in 389 patients (92.6%), followed by MTC, which was found in 21 patients (5%). Unifocal tumors were dominant, found in 211 patients (50.2%). Unifocal unilateral tumors represented 64% of the patients, in which the tumor was confined to one thyroid lobe. About two-thirds (65%) of the patients showed pathologically positive central lymph nodes, and most of them (87%) showed positive lateral lymph nodes as well. Early T-stages (i.e., T1 and T2) tumors represented 68%, while only 4% had locally advanced (T4) tumors.

### Complications (Table 3)

**Table 3:** Intraoperative and postoperative complications.

Variable	Value
Intraoperative complications	
No	373 (88.8%)
Yes	47 (11.2%)
Type of Intraoperative complication	
Bleeding	1 (0.2%)
IJV injury	14 (3.3%)
Recurrent laryngeal nerve injury/sacrifice	16 (3.8%)
Tracheal injury	1 (0.2%)
Stridor	5 (1.2%)
Spinal accessory nerve injury/sacrifice	3 (0.7%)
Chyle leak	1 (0.2%)
Pharyngeal injury	3 (0.7%)
Marginal mandibular nerve injury	2 (0.5%)
Sacrifice of IJV, Vagus, SCM	1 (0.2%)
Postoperative complications	
No	317 (73.1%)
Yes	113 (26.9%)
Postoperative complications#	
Hypocalcemia	69 (16.4%)
Nerve affection	35 (8.3%)
Bleeding	4 (1%)
Chyle leak	6 (1.4%)
Tracheostomy	5 (1.2%)
Hoarseness of voice	5 (1.2%)
Choking	3 (0.7%)
Seroma/collection	6 (1.4%)
Hypocalcemia type*	
Temporary	54 (79.4%)
Permanent	14 (20.6%)
Readmission for hypocalcemia	21 (5%)
Type of nerve affection*	
Temporary	18 (54.5%)
Permanent	15 (45.5%)

* Valid percent.

# Some patients have more than 1 complication.

Out of 420 procedures, intraoperative complications were encountered in 47 (11.2%). The commonest was recurrent laryngeal nerve injury, which occurred in 16 patients, representing 34% of the morbidities. In eight patients (out of the 16), the recurrent laryngeal nerve was intentionally sacrificed as it was infiltrated. Stridor was detected immediately after extubating five patients (10.6% of the intraoperative complications).

The commonest postoperative morbidity was hypocalcemia (16.4%), twenty-one patients (5%) required hospital re-admission for calcium intravenous infusion. Recurrent laryngeal nerve paralysis manifested in thirty-five patients (8.3%), which mandated tracheostomy in five, then chyle leak and seroma/collection in six patients (1.4%) each.

For the long-term sequelae, most of the hypocalcemic events were resolved (79.4%), while a fifth of the patients (20.6%) suffered from permanent hypocalcemia. And out of 35 postoperative recurrent laryngeal nerve affections, 15 manifestations persisted, with more than half of the affections (54.5%) resolved. The total number of permanent nerve affections (15 injuries) represented roughly the actual incidents of nerve injury (16 injuries) (i.e., only one missed injury).

### Predictors of hypocalcemia (Table 4)

**Table 4:** Predictors of postoperative hypocalcemia.

	Univariate			Multivariate	
	Hypocalcemia		P-value	OR	Significance
	No	Yes			
Sex			0.058		
Male	105	13			
Female	245	57			
Age mean +/- SD years	39.9 +/- 15.2	37.5 +/- 16.4	0.23		
BMI median (range) Kg/m2	30.5 (17.5–56.9)	35.2 (21–54.7)	**0.008**	1.03	0.22
Pathology			0.29		
PTC	320	69			
MTC	21	0			
FTC	5	0			
HTC	1	0			
ATC	1	0			
SETTLE	1	0			
Central node dissection			**0.029**	1.3	0.49
Ipsilateral	231	36			
Bilateral	119	34			
Pattern of nodal dissection			0.65		
Prophylactic	106	22			
Therapeutic	187	40			
Sampling	36	4			
Excised with specimen	14	2			
Operative time median (range) minutes	180 (60–540)	240 (90–600)	**< 0.001**	1	0.17
Pathologic focality			0.69		
Unifocal	178	33			
Multifocal	162	34			
Pathologic laterality			**0.034**	1.6	0.23
Unilateral	233	36			
Bilateral	109	31			
Extrathyroid extension			0.34		
No	210	34			
Yes	91	20			
T stage			0.24		
1	129	18			
2	114	24			
3	84	18			
4	12	5			
Central node status			0.214		
Negative	122	19			
Positive	223	51			
Pathologic tumour size median (range) cm	2.5 (0.1-8)	3 (0.5-8)	0.063		
Central node harvest median (range)	5 (1–35)	6 (1–25)	0.31		
Positive central node count median (range)	3 (1–27)	3 (1–17)	0.72		
Length of hospital stay	2 (1–19)	4 (1–25)	**< 0.001**	**1.2**	**0.001**

High body mass index (BMI) (p-value = 0.008), bilateral CLND (p-value = 0.029), prolonged operative time (p-value < 0.001), and tumor bilaterality (p-value = 0.034) were significantly associated with postoperative hypocalcemia. However, interestingly, neither the positivity of central nodes (p-value = 0.21) nor the number of harvested central nodes (p-value = 0.31) was correlated with hypocalcemia.

### Predictors of recurrent laryngeal nerve affection (Table 5)

**Table 5:** Predictors of postoperative nerve affection.

Variable	Univariate			Multivariate	
	Recurrent laryngeal nerve injury		P-value	OR	Significance
	No	Yes			
Sex			0.43		
Male	105	12			
Female	279	23			
Age mean +/- SD years	39.2 +/- 14.9	41.9 +/-18.8	0.301		
BMI median (range) Kg/m2	30.9 (17.5–56.9)	34.9 (18.9–54.7)	0.17		
Pathology			**0.019**		1
PTC	354	34			
MTC	21	0			
FTC	5	0			
HTC	1	0			
ATC	0	1			
SETTLE	1	0			
Central node dissection			0.27		
Ipsilateral	248	19			
Bilateral	136	16			
Pattern of nodal dissection			0.22		
Prophylactic	122	6			
Therapeutic	201	25			
Sampling	37	3			
Excised with specimen	15	1			
Operative time median (range) minutes	180 (60–600)	240 (90–540)	**0.016**	0.42	1
Pathologic focality			0.16		
Unifocal	197	14			
Multifocal	174	21			
Pathologic laterality			**0.039**	1.7	0.23
Unilateral	252	17			
Bilateral	121	18			
Extrathyroidal extension			**0.026**	0.83	0.74
No	228	16			
Yes	94	16			
T stage			**< 0.001**		0.46
1	142	5			
2	126	12			
3	90	12			
4	11	6			
Central node status			0.092		
Negative	134	7			
Positive	245	28			
Pathologic tumour size median (range) cm	2.5 (0.1-8)	3 (0.5-7)	**0.002**	1.1	0.44
Central node harvest median (range)	5 (1–35)	4.5 (1–25)	0.89		
Positive central node count median (range)	3 (1–27)	4 (1–17)	0.38		
Length of hospital stay	2 (1–23)	4 (1–25)	**< 0.001**	1.2	**0.012**

Pathologic type (papillary and anaplastic) (p-value = 0.019), prolonged operative time (p-value = 0.016), tumor bilaterality (p-value = 0.039), extrathyroidal extension (p-value = 0.026), the advanced T stage (p-value < 0.001), and the larger tumor size (p-value = 0.002) were significantly associated with postoperative nerve paralysis. However, again, neither the positivity of central nodes (p-value = 0.092) nor the number of harvested central nodes (p-value = 0.89) was associated with nerve affection.

Both hypocalcemia and recurrent laryngeal nerve affection were associated with prolonged hospital stay.

## Discussion

CLND (either prophylactic or therapeutic) is recommended with thyroidectomy for patients of high-risk DTC and along with LLND for patients of MTC, according to the latest guidance^3,8^.

The detection rate of central compartment involvement was limited in the pre-operative sonographic modalities (8.3%) in our study, which is consistent with the Yang et al. findings^12^. This raised concerns about the need for developing novel diagnostic tools incorporating AI-based algorithms (i.e., *radiomics*), designing nomograms and prediction models of CLNM^13–15^.

This retrospective review showed various modes of management of the central compartment, in which the most common approach was the ipsilateral clearance of the central nodes, based on the pre-operative data confirming the unilaterality of the disease (i.e. involving one thyroid lobe), the relatively low-risk of contralateral CLNM (2–20% in different series), and the indeterminate oncologic outcomes^16^. While bilateral central dissection was carried out in the setting of bi-lobar disease or heavy nodal burden, or medullary pathology. This could be explained by the current body of evidence^17–20^ and our analysis, showing an increased risk of permanent hypocalcemia in bilateral disease (*p* = 0.034) and bilateral CLND (*p* = 0.029).

Our data linked the incidence of postoperative hypocalcemia to the extended operative times, which may be explained by the extensive dissection of the central compartment and manipulation of the parathyroid tissue. Hypocalcemia was significantly associated with high BMI, which seems plausible with the difficult fatty central compartment.

Parathyroid glands are usually marked by a stitch upon commencement of central dissection to avoid inadvertent removal with the lymph packet, but this technique is not yet fail-proof. New techniques were developed to help parathyroid gland identification, the most recent is Near-Infra-Red Auto-Fluorescence (NIRAF), which uses endogenous fluorophores within the parathyroids that can be lit by a 785 nm laser light, allowing the surgeon to “see” the glands within the lympho-fatty tissue. This technique was applied and approved by the Food and Drug Administration (FDA), with promising success rates in avoiding hypocalcemia^21^.

Results showed that the risk of recurrent laryngeal nerve injury increased mainly with more advanced T-stage and tumor bilaterality. Neither the nodal burden nor the type of central dissection affected recurrent laryngeal nerve injury, this is consistent with the published literature negating the effect on the central nodes and the dissection on the recurrent laryngeal nerve integrity^7^. And coping with our findings, the operative time seemed to be extended when nerve injury/sacrifice was encountered, reflecting the difficult field in such cases.

Our study has some limitations. Because of being a retrospective study, some data were missing. Surgical techniques also were not unified. Intraoperative nerve monitoring is not available in our center. Preoperative serum calcium, parathormone hormone, and vitamin D levels were not monitored for postoperative comparison, which may have given valuable data about the patient’s metabolic profile. On the other hand, what strengthens our study is that all these data are prospectively registered on our institutional database, and the number of included patients is large.

## Conclusion

CLND for thyroid cancers has a significant morbidity, affecting nearly 25% of the patients. Hypocalcemia followed by recurrent laryngeal nerve paralysis is the most common. Bilateral CLND and obesity are independent predictors of hypocalcemia, while advanced T stage, extra-thyroid extension, bilaterality, and pathologic type are predictors of nerve affection. Fortunately, about 80% of hypocalcemia and more than half of nerve affection symptoms will resolve with conservative measures. Better diagnostic tools and predictive models are mandatory to identify central compartment disease burden, bilaterality, and significance. Moreover, more advanced surgical techniques are needed to spare the patients the potential complications.

## Electronic supplementary material

Below is the link to the electronic supplementary material.

Supplementary Material 1

## Data Availability

All the data used are available on request from the corresponding author.
