# Correction: Morbidity patterns and long-term outcomes of central lymph node dissection in thyroid cancer patients

**DOI:** 10.1038/s41598-025-19577-4

**Published:** 2025-09-17

**Authors:** Khalid Atallah, Shadi Awny, Khaled Abdelwahab, Ahmed Abdallah, Islam H Metwally, Omar Hamdy, Mohammed Zuhdy, Ahmed Fareed

**Affiliations:** 1 Surgical Oncology Department, Oncology Center, Faculty of Medicine, Mansoura University, Mansoura, Egypt; 2 Surgical Oncology Department, King Abdullah Medical City, Makkah, Kingdom of Saudi Arabia

Correction to: *Scientific Reports* 10.1038/s41598-025-08439-8, published online 02 July 2025

The original version of this Article contained errors in the Reference list, where Reference 3 was incorrectly included while not cited in the text. Reference 8 should have been listed as Reference 3, the intended Reference 8 was omitted, and References 12–21 were omitted.

As a result, in the Discussion section, Reference citations were incorrect, where

“CLND (either prophylactic or therapeutic) is recommended with thyroidectomy for patients of high-risk DTC and along with LLND for patients of MTC, according to the latest guidance^3,11^.

The detection rate of central compartment involvement was limited in the pre-operative sonographic modalities (8.3%) in our study, which is consistent with the Yang et al. findings (14). This raised concerns about the need for developing novel diagnostic tools incorporating AI-based algorithms (i.e., *radiomics*), designing nomograms and prediction models of CLNM (15–17).

This retrospective review showed various modes of management of the central compartment, in which the most common approach was the ipsilateral clearance of the central nodes, based on the pre-operative data confirming the unilaterality of the disease (i.e. involving one thyroid lobe), the relatively low-risk of contralateral CLNM (2–20% in different series), and the indeterminate oncologic outcomes (18). While bilateral central dissection was carried out in the setting of bi-lobar disease or heavy nodal burden, or medullary pathology. This could be explained by the current body of evidence (19–22) and our analysis, showing an increased risk of permanent hypocalcemia in bilateral disease (*p* = 0.034) and bilateral CLND (*p* = 0.029). […]

This technique was applied and approved by the Food and Drug Administration (FDA), with promising success rates in avoiding hypocalcemia (23). […].

Neither the nodal burden nor the type of central dissection affected recurrent laryngeal nerve injury, this is consistent with the published literature negating the effect on the central nodes and the dissection on the recurrent laryngeal nerve integrity (24).”

now reads:

“CLND (either prophylactic or therapeutic) is recommended with thyroidectomy for patients of high-risk DTC and along with LLND for patients of MTC, according to the latest guidance^3,8^.

The detection rate of central compartment involvement was limited in the pre-operative sonographic modalities (8.3%) in our study, which is consistent with the Yang et al. findings^12^. This raised concerns about the need for developing novel diagnostic tools incorporating AI-based algorithms (i.e., *radiomics*), designing nomograms and prediction models of CLNM^13–15^.

This retrospective review showed various modes of management of the central compartment, in which the most common approach was the ipsilateral clearance of the central nodes, based on the pre-operative data confirming the unilaterality of the disease (i.e. involving one thyroid lobe), the relatively low-risk of contralateral CLNM (2–20% in different series), and the indeterminate oncologic outcomes^16^. While bilateral central dissection was carried out in the setting of bi-lobar disease or heavy nodal burden, or medullary pathology. This could be explained by the current body of evidence^17–20^ and our analysis, showing an increased risk of permanent hypocalcemia in bilateral disease (*p* = 0.034) and bilateral CLND (*p* = 0.029). […]

This technique was applied and approved by the Food and Drug Administration (FDA), with promising success rates in avoiding hypocalcemia^21^. […]

Neither the nodal burden nor the type of central dissection affected recurrent laryngeal nerve injury, this is consistent with the published literature negating the effect on the central nodes and the dissection on the recurrent laryngeal nerve integrity^7^.”

As a result, the Reference list now reads:

1. Sun, W. et al. Risk factors for central lymph node metastasis in CN0 papillary thyroid carcinoma: A systematic review and meta-analysis. *PLoS ONE*. **10**(10), e0139021 (2015).
2. Cabanillas, M. E., McFadden, D. G. & Durante, C. Thyroid cancer. *Lancet*. **388**(10061), 2783–2795 (2016).
3. Haugen, B. R. et al. 2015 American Thyroid Association management guidelines for adult patients with thyroid nodules and differentiated thyroid cancer: The American Thyroid Association guidelines task force on thyroid nodules and differentiated thyroid cancer. *Thyroid*. **26**(1), 1–133 (2016).
4. Kouvaraki, M. A. et al. Role of preoperative ultrasonography in the surgical management of patients with thyroid cancer. *Surgery*. **134**(6), 946–954 (2003).
5. White, M. L., Gauger, P. G. & Doherty, G. M. Central lymph node dissection in differentiated thyroid cancer. *World J. Surg.* **31**, 895–904 (2007).
6. Seok, J. et al. Factors affecting central node metastasis and metastatic lymph node ratio in papillary thyroid cancer. *Otolaryngol.–Head Neck Surg.* **165**(4), 519–527 (2021).
7. Giordano, D. et al. Complications of central neck dissection in patients with papillary thyroid carcinoma: Results of a study on 1087 patients and review of the literature. *Thyroid*. **22**(9), 911–917 (2012).
8. National Comprehensive Cancer Network, Inc. All rights reserved. Accessed [4.2024]. Comprehensive clinical practice guidelines published by the National Comprehensive Cancer Network regarding the work-up, management and follow-up for thyroid cancer (2024).
9. Baud, G. et al. Impact of lymph node dissection on postoperative complications of total thyroidectomy in patients with thyroid carcinoma. *Cancers*. **14**(21), 5462 (2022).
10. de Jong, M. et al. Children are at a high risk of hypocalcaemia and hypoparathyroidism after total thyroidectomy. *J. Pediatr. Surg.* **55**(7), 1260–1264 (2020).
11. Kazaure, H. S. et al. Severe hypocalcemia after thyroidectomy: An analysis of 7366 patients. *Ann Surg.* **274**(6), e1014–e1021 (2021).
12. Yang, J., Zhang, F. & Qiao, Y. Diagnostic accuracy of ultrasound, CT and their combination in detecting cervical lymph node metastasis in patients with papillary thyroid cancer: A systematic review and meta-analysis. *BMJ Open.* **12**(7), e051568 (2022).
13. Tong, Y. et al. Ultrasound-based radiomics analysis for preoperative prediction of central and lateral cervical lymph node metastasis in papillary thyroid carcinoma: A multi-institutional study. *BMC Med. Imaging*. **22**(1), 82 (2022).
14. Li, J. et al. Computed tomography-based radiomics model to predict central cervical lymph node metastases in papillary thyroid carcinoma: A multicenter study. *Front. Endocrinol.* **12**, 741698 (2021).
15. Feng, J. W. et al. A nomogram based on clinical and ultrasound characteristics to predict central lymph node metastasis of papillary thyroid carcinoma. *Front. Endocrinol.* **12**, 666315 (2021).
16. Chen, L. et al. Prophylactic central neck dissection for papillary thyroid carcinoma with clinically uninvolved central neck lymph nodes: A systematic review and meta‐analysis. *World J. Surg.* **42**(9), 2846–2857 (2018).
17. Back, K., Choe, J. H., Kim, J. S. & Kim, J. H. Occult contralateral central neck metastasis in papillary thyroid carcinoma with preoperatively documented ipsilateral lateral neck metastasis. *Eur. J. Surg. Oncol.* **47**(6), 1339–1345 (2021).
18. Hartl, D. M. et al. Risk staging with prophylactic unilateral central neck dissection in low-risk papillary thyroid carcinoma. *Eur. J. Surg. Oncol.* **49**(3), 568–574 (2023).
19. Kim, D. H., Kim, G. J., Kim, S. W. & Hwang, S. H. Predictive value of ipsilateral central lymph node metastasis for contralateral central lymph node metastasis in patients with thyroid cancer: Systematic review and meta‐analysis. *Head Neck*. **43**(10), 3177–3184 (2021).
20. Hughes, D. T. et al. Prophylactic central compartment neck dissection in papillary thyroid cancer and effect on locoregional recurrence. *Ann. Surg. Oncol.* **25**, 2526–2534 (2018).
21. Guerlain, J. et al. Intraoperative parathyroid gland identification using autofluorescence imaging in thyroid cancer surgery with central neck dissection: Impact on post-operative hypocalcemia. *Cancers*. **16**(1), 182 (2023).

The original Article has been corrected.

