# Supplementary material for: Morbidity patterns and long-term outcomes of central lymph node dissection in thyroid cancer patients
Source: Sci Rep. 2025 Jul 2;15:23527. doi: 10.1038/s41598-025-08439-8 (PMC12222766; doi:10.1038/s41598-025-08439-8)
Supplement: Supplementary file 1 — Supplementary Material 1 [file 41598_2025_8439_MOESM1_ESM.pptx]

## Slide 1
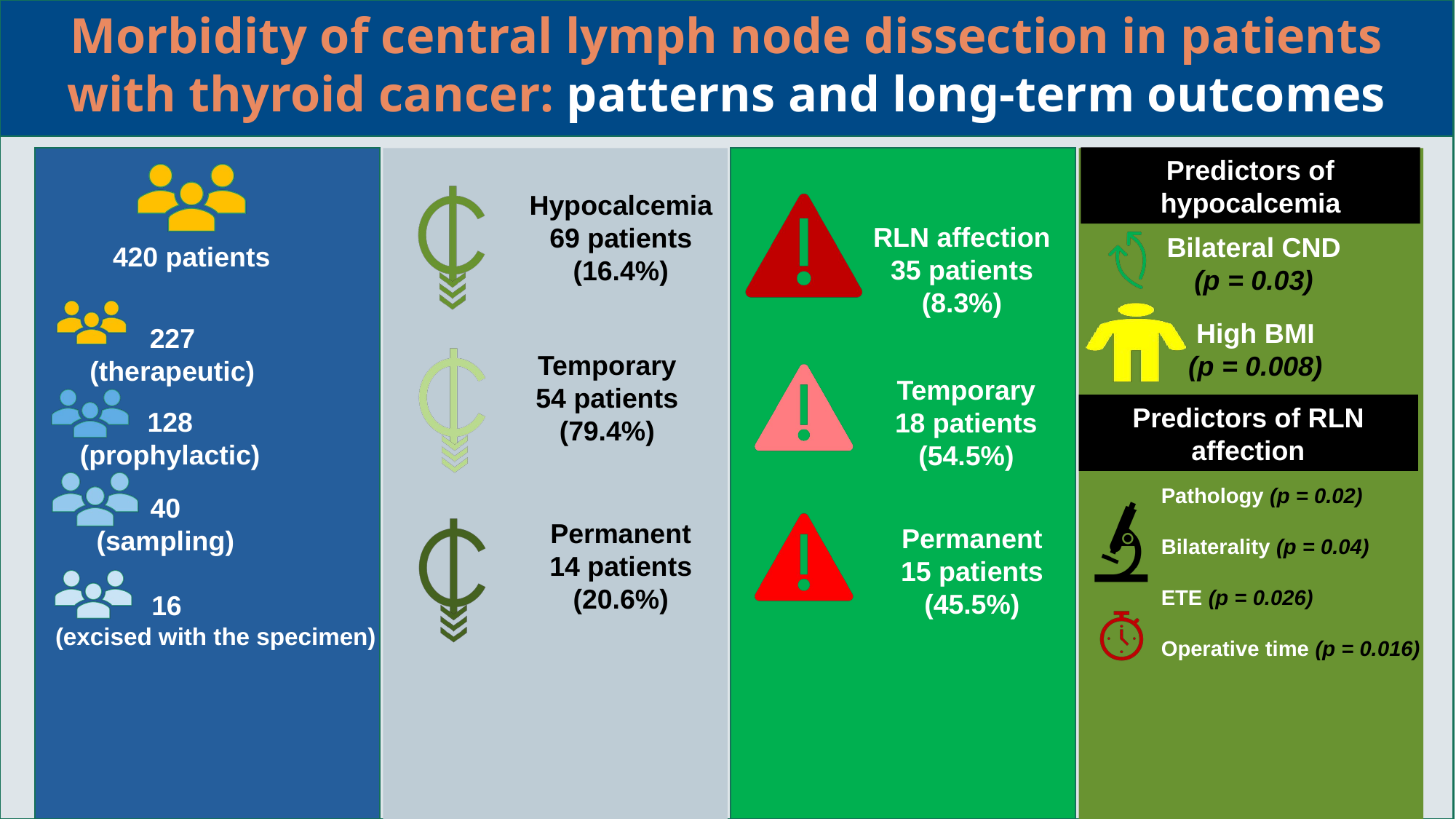

Morbidity of central lymph node dissection in patients with thyroid cancer: patterns and long-term outcomes
Predictors of hypocalcemia
Hypocalcemia
69 patients
(16.4%)
RLN affection
35 patients (8.3%)
Bilateral CND
(p = 0.03)
420 patients
High BMI
(p = 0.008)
227
(therapeutic)
Temporary
54 patients
(79.4%)
Temporary
18 patients (54.5%)
Predictors of RLN affection
128
(prophylactic)
Pathology (p = 0.02)
Bilaterality (p = 0.04)
ETE (p = 0.026)
Operative time (p = 0.016)
40
(sampling)
Permanent
14 patients
(20.6%)
Permanent
15 patients (45.5%)
 16
 (excised with the specimen)
